# Association Between Retinoid X Receptor Gene Variants and Dyslipidemia Risk in an Iranian Population

**DOI:** 10.7759/cureus.17730

**Published:** 2021-09-05

**Authors:** Farzane Vafaeie, Toba Kazemi, Saeede Khosravi, Ebrahim Miri Moghaddam

**Affiliations:** 1 Genetics, Cardiovascular Diseases Research Center, Birjand University of Medical Sciences, Birjand, IRN; 2 Cardiology, Cardiovascular Diseases Research Center, Birjand University of Medical Sciences, Birjand, IRN; 3 Epidemiology and Public Health, Cardiovascular Diseases Research Center, Birjand University of Medical Sciences, Birjand, IRN

**Keywords:** dyslipidemia, single nucleotide polymorphism, t-arms pcr, retinoid x receptor, clinical characteristics

## Abstract

Background

Dyslipidemia is a complex trait that is influenced by various genetic and environmental factors. While the exact cause of dyslipidemia is still unknown, some studies have shown that genetic factors such as single nucleotide polymorphisms (SNPs) have been primarily associated with dyslipidemia. Based on the available data, it appears that retinoid X receptor (RXR) genes are jointly or separately associated with lipid homeostasis and that SNPs may affect RXR gene functions in lipid metabolism.

Methods

To study the possible role of the RXR genes in genetic susceptibility of dyslipidemia, three selected polymorphisms, rs3132294 located in RXRA (RXR-alpha) gene and rs2651860 and rs1128977 located in RXRG (RXR-gamma) gene, were investigated in 391 individuals with the use of tetra-primer amplification refractory mutation system polymerase chain reaction (T-ARMS PCR) method.

Results

For the rs3132294 SNP, the genotype frequencies in the case group were GG 58.5%, GA 33.2%, and AA 8.3%, and in the control group, they were GG 51.8%, GA 36.3%, and AA 11.9%. The genotype distribution of rs2651860 SNP in the case group were TT 43.2%, TG 52.1%, and GG 4.7%, and in the control group, they were TT 50.8%, TG 46.2%, and GG 3%. Genotype frequencies for the rs1128977 SNP in the case group were CC 34.7%, CT 47.6% and TT 17.7%, compared with CC 37.8%, CT 44.3%, and TT 17.9% in the control group. When the clinical characteristics of the case and control groups were stratified by allele carrier status for each SNP, the rs1128977 SNP was associated with increased levels of HDL-cholesterol, body mass index, waist circumference, and diastolic blood pressure (P< 0.05). In contrast, the alleles of the rs2651860 and rs3132294 SNP were not associated with an increased prevalence of dyslipidemia or clinical characteristics in the case group compared to the control group.

Conclusion

The present study suggests that rs1128977 SNP in the RXRG gene may affect the clinical characteristics in cases. However, further genetics association studies on large samples are required to validate our findings.

## Introduction

Dyslipidemia is a complex and multifactorial disorder that can be influenced by various genetic and environmental factors [1]. Dyslipidemias are revealed by a decrease concentration of high-density lipoprotein-cholesterol (HDL-C), insufficient reduction in low-density lipoprotein-cholesterol (LDL-C) concentration, the elevation of triglyceride (TG) and total cholesterol (TC) in the serum blood [2, 3]. Since hyperlipidemia is a well-established risk factor for stroke, hypertension, and coronary artery disease (CAD), it is a major public health concern in many countries. The prevalence of dyslipidemia varies widely worldwide. Available studies indicated the prevalence of dyslipidemia in individuals aged ≥ 15 in Iranian people was 41.6% for hypercholesterolemia (≥200 mg/dl), 46.0% for hypertriglyceridemia (≥150 mg/dl), 35.5% for LDL-C (≥ 130 mg/dl) and 43.9% for HDL-C (<40 mg/dl in males, <50 mg/dl in females) [4].

While the exact cause of dyslipidemia remains unknown, previous investigations have indicated that genetic variation strongly influences individual lipid proﬁle [5]. The candidate gene approach and linkage-analysis have identified several candidate genes and loci for susceptibility to this lipid abnormality [3]. To date, a large number of single nucleotide polymorphisms (SNPs) and mutations have been associated with dyslipidemia [6]; however, they do not entirely explain all the variabilities; therefore, the research for additional candidate genes and variations continues.

Nuclear receptors (NR) are members of a large superfamily of evolutionarily related transcription factors (TFs) that can be stimulated by specific ligands. These receptors are involved in regulating gene expression in a plethora of physiological, environmental, and developmental processes, cell differentiation, cell death, and various metabolic pathways [7].

The retinoid X receptor (RXR) is a nuclear receptor that functions as a ligand-activated transcription factor induced by 9-cis retinoic acid. RXR consists of three isoforms, including RXR-alpha (RXRA), RXR-beta (RXRB), and RXR-gamma (RXRG) [8]. RXRs have effects on various pathways due to their ability to regulate the transcription of target genes. RXRs create either homodimers or heterodimers with other nuclear receptors comprising the liver X receptors (LXRs), pregnane X receptor (PXR), retinoic acid receptors (RARs), peroxisome proliferator-activated receptors (PPARs), farnesoid X receptor (FXRs), thyroid hormone receptor (TR), constitutive androstane receptor (CAR) and vitamin D receptor (VDR). RXRs are an obligatory partner in FXRs, LXRs, and PPARs activation that are involved in the regulation of genes in triglyceride and cholesterol metabolism [2, 9]. Moreover, recent evidence has revealed that PXR and CAR influence lipid metabolism and homeostasis [8]. Furthermore, linkage analysis in dizygotic twins and their parents has shown that RXR genes are associated with LDL, TG, and TC concentrations [10, 11]. Hence, it can be concluded that the RXR genes are jointly or separately related to lipid homeostasis.

We hypothesized that SNPs in the RXR genes might be associated directly or indirectly with dyslipidemia. To our knowledge, few studies have investigated the effect of variants in the RXR genes on blood lipid and lipoprotein levels. We aimed to evaluate the association between rs2651860, rs1128977, and rs3132294 SNPs in the RXR genes and dyslipidemia in the Iranian population in the present study.

## Materials and methods

Study population

This case-control study was performed with 391 individuals (195 cases with dyslipidemia and 196 healthy randomized controls) referred to Razi Hospital in Birjand city from April 2018 to June 2019. Informed consent was obtained from the participants before the study. Routine biochemical data, including fasting blood sugar (FBS), total cholesterol (TC), triglyceride (TG), HDL-C, and LDL-C, were examined after overnight fasting. BMI, waist circumference, systolic blood pressure, and diastolic blood pressure were measured. The cases were required to have TG, or TC levels greater than or equal to the 90th percentile (TG or/and TC ≥ 250 mg/dl). The control subjects were unrelated and randomly selected from normolipidemic individuals. The exclusion criteria were: FBS ≥ 125 mg/dl, TG ≥ 200 mg/dl, TC ≥ 200 mg/dl, LDL-C ≤ 130 mg/dl, and HDL-C ≥ 50 mg/dl for women, and HDL ≥ 40 mg/dl for men. Serum LDL-C, HDL-C, TG, and TC levels were directly measured by the enzymatic method using a Hitachi 7600 auto-analyzer (Hitachi, Tokyo, Japan). FBS was measured with a glucose oxidase method. A total of nine individuals with serious complications were excluded from the study. The exclusion criteria were hepatic disease (patients with positive tests for chronic hepatitis C and B), kidney disease (patients with serum creatinine levels of > 1.5 mg/dl), thyroid disease (patients with a history of hypothyroidism [TSH > 4.0/mU/L], or hyperthyroidism [TSH < 0.5 mU/L]), pregnancy, type 1 diabetes mellitus, and maturity-onset diabetes of the young (MODY), cardiovascular disease (patients with valvular disease, elevated serum cardiac biomarkers, and ECG changes) and patients taking medication for stroke and hypertension.

DNA extraction and genotype analysis

Genomic DNA was extracted by the manual salting-out method from 2 ml of peripheral blood samples containing EDTA (1.8 mg EDTA per milliliter of blood) [12]. The quality and quantity of extracted DNA were measured using Epoch™ microplate spectrophotometer (Biotek Instrument, USA).

The SNP genotyping of rs2651860 T > G (RXRG gene, chromosome 1q), rs1128977 C > T (*RXRG* gene, chromosome 1q), and rs3132294 G > A (RXRA gene, chromosome 9q) were carried out by the T-ARMS PCR method using two pairs of primers (two outers and two inners primers) listed in Table 1.

**Table 1:** Primer sequences and PCR fragments of RXRG and RXRA variants.

Gene	SNP	The sequence of forward (F) and reverse (R) primers	Product size (bp)
RXRA	rs3132294	F outer: 5'-AGCCCCAAGCCCTGCTCTCCT-3'	Outers: 387, G allele: 238, A allele: 194
		R outer: 5'-TCCCACCCTCCTTGAGCTCAGTG-3'	
		F inner: 5'-TCCGACGCACAGTGATGAACAATG-3'	
		R inner: 5'-CGTCTGGGCTCCGCGGTTAAT-3'	
RXRG	rs2651860	F outer: 5'-TAGGAGCTGCTAGTGGAGTAGCTGT-3'	Outers: 422, T allele: 271, G allele: 205
		R outer: 5'-GGGTATTTAGTAAGTGAAGGGGTGG-3'	
		F inner: 5'-AGTCTGCTTTCAATCCTAATGTATAGATAG-3'	
		R inner: 5'-CAGTGCAACCTCAGAACCCTTСА-3'	
RXRG	rs1128977	F outer: 5'-TGTTTTTTCTTGAGTAAGCCCTCATTGC-3'	Outers: 382, C allele: 197, T allele: 241
		R outer: 5'-CCCAGTGACATAAACGTATAGGTGGGTC-3'	
		F inner: 5'-CGGATCTCTGGTTAAACACATCTGTTCC-3'	
		R inner: 5'-TGTACCTGAGGATCTGTCTCCACAGCTA-3'	

The PCR amplification was carried out on a BioRad thermocycler (BioRad Laboratories Inc., Hercules, CA, USA) in the final volume of 20 μl containing 100 ng genomic DNA, 10 μl Taq DNA polymerase Master Mix RED (Ampliqon, Odense, Denmark), and 10 pmol of each pair of primers along with sterile distilled water up to 20 μl. PCR amplifications were performed with an initial denaturation at 95 °C for five minutes. This followed by 30 cycles, including denaturation at 95˚ C for 30 s and 40 s annealing at 61 °C for rs3132294 SNP, 59 °C for rs2651860 SNP, 63 °C for rs1128977 SNP and 45 s extension at 72 °C, followed by a final extension at 72 °C for seven minutes. Afterwards, the PCR products were evaluated by electrophoresis on 1.5% agarose gel, stained by FluoroVue nucleic acid gel stain (SMOBIO, Hsinchu, Taiwan), and visualized under ultraviolet light (Molecular Imager Gel Doc XR System, BioRad, Hercules, California).

Statistical analysis

In this investigation, the normal distribution of variables was appraised by using the Kolmogorov-Smirnov normality test. The chi-square test and Mann-Whitney U test (or independent two-sample Student t-test) were used to compare the difference between case and control groups for nominal and numerical variables, respectively. Demographic characteristics were examined in case and control groups. Data were represented as mean ± standard deviation and numbers (%) for numerical and nominal variables. After adjustment for covariates, multivariate logistic regression was recomputed to assess the association between dyslipidemia and genotypes. A 95% conﬁdence interval (CI) and odds ratio were used to evaluate the association between variables. All statistical calculations were done by SPSS Statistics software v.19 (IBM Corp., Armonk, NY). A p-value less than 0.05 was declared statistically signiﬁcant.

## Results

Participants had a mean age of 41.27 ± 11.79 years, 49.5% female and 50.5%, male. Table 2 demonstrates the demographic characteristics of study groups. Analyses in cases showed significantly lower HDL-C (P<0.001), higher TC (P<0.001), LDL-C (P<0.001), and TG (P<0.001) compared to controls, as expected. Also, significant differences in the body mass index, waist circumference, diastolic blood pressure, systolic blood pressure, and FBS levels have been detected between cases and controls.

**Table 2:** Laboratory and demographic characteristics of the study groups Data are presented as mean ± standard deviation or n (%) unless otherwise stated. ^a^ t-test, ^b^ Mann-Whitney, ^* ^statistically significant

Characteristics	Total (n=391)	Control (n=196)	Case (n=195)	P-value	test statistics
Gender (Male)	50.5%	56%	44.5%	0.039^*^	Pearson Chi-Square=4.246
Age (Years)^ a^	41.25±11.82	36.67±10.19	45.90±11.58	<0.001^*^	t=6.983
Body mass index (kg/m^2^)^ a^	25.71±4.74	23.53±4.40	27.80±4.08	<0.001^*^	t=8.546
Waist circumference (cm)^a^	91.65±13.17	85.48±12.41	97.72±10.89	<0.001^*^	t=8.781
Systolic blood pressure (mmHg)^ b^	116.65±11.77	115.07±11.39	118.25±11.99	0.007^*^	Z =-2.700
Diastolic blood pressure (mmHg)^ b^	77.09±7.42	76.07±7.87	78.13±6.79	0.017^*^	Z =-2.387
Fasting blood sugar (mg/dl)^ b^	93.08±8.62	91.73±7.38	94.36±9.50	0.026^*^	Z =-2.227
Total cholesterol (mg/dl) ^b^	209.64±62.35	166.46±23.94	250.78±59.81	<0.001^*^	Z =-13.207
Triglyceride (mg/dl)^ b^	163.04±123.61	81.16±30.90	241.05±128.33	<0.001^*^	Z =-12.541
HDL-cholesterol (mg/dl)^ b^	44.63±12.93	52.15±9.14	37.47±11.92	<0.001^*^	Z =-10.707
LDL-cholesterol (mg/dl)^ b^	122.80±50.66	94.87±18.71	149.41±56.86	<0.001^*^	Z =-10.596

Association analyses and the logistic regression model after adjustment for rs3132294 G > A variant were reported in Table 3. The frequency of the genotypes and alleles showed that there were no significant differences in cases and controls.

**Table 3:** The frequency of the genotypes and alleles of rs3132294 G > A

RXRA rs3132294 G > A	Total (n=391)	Control (n=196)	Case (n=195)	OR (95% CI)	P-value
Codominant					
GG	55.2%	51.8%	58.5%	1 (Ref)	-
GA	34.7%	36.3%	33.2%	0.809 (0.525-1.247)	0.337
AA	10.1%	11.9%	8.3%	0.616 (0.308-1.230)	0.170
Allele					
G	72.5%	69.9%	75.1%	1 (Ref)	-
A	27.5%	30.1%	24.9%	0.771 (0.561-1.058)	0.107
Dominant					
GG	55.2%	51.8%	58.5%	1 (Ref)	-
AA+GA	44.8%	48.2%	41.5%	0.761 (0.509-1.138)	0.183
Recessive					
GA+GG	89.9%	88.1%	91.7%	1 (Ref)	-
AA	10.1%	11.9%	8.3%	0.668 (.341-1.308)	0.237
Over-dominant					
GG+AA	65.3%	63.7%	66.8%	1 (Ref)	-
GA	34.7%	36.3%	33.2%	0.872 (0.573-1.326)	0.521

The same analyses were performed for rs2651860 and rs1128977 SNPs (Tables 4-5). Again, individuals carrying the variants showed no statistically significant differences.

**Table 4:** The frequency of the genotypes and alleles of rs2651860 T > G

RXRG rs2651860 T > G	Total (n=391)	Control (n=196)	Case (n=195)	OR (95% CI)	P-value
Codominant					
TT	47%	50.8%	43.2%	1 (Ref)	-
GT	49.1%	46.2%	52.1%	1.324 (0.882-1.988)	0.176
GG	3.9%	3%	4.7%	1.807 (0.618-5.286)	0.280
Allele					
T	71.6%	73.9%	69.3%	1 (Ref)	
G	28.4%	26.1%	30.7%	1.253 (0.917-1.713)	0.156
Dominant					
TT	47%	50.8%	43.2%	1 (Ref)	-
GG+GT	53%	49.2%	56.8%	1.354 (0.908-2.019)	0.137
Recessive					
GT+TT	96.1%	97%	95.3%	1 (Ref)	-
GG	3.9%	3%	4.7%	1.566 (0.546-4.486)	0.400
Over-dominant					
TT+GG	50.9%	53.8%	47.9%	1 (Ref)	-
GT	49.1%	46.2%	52.1%	1.266 (0.850-1.886)	

**Table 5:** The frequency of the genotypes and alleles of rs1128977 C > T

RXRG rs1128977 C > T	Total (n=391)	Control (n=196)	Case (n=195)	OR (95% CI)	P-value
Codominant					
CC	36.3%	37.8%	34.7%	1 (Ref)	-
CT	45.9%	44.3%	47.6%	1.155 (0.742-1.798)	0.522
TT	17.8%	17.9%	17.7%	1.073 (0.603-1.909)	0.811
Allele					
C	59.3%	59.9%	58.6%	1 (Ref)	-
T	40.7%	40.1%	41.4%	1.058 (0.794-1.409)	0.701
Dominant					
CC	36.3%	37.8%	34.7%	1 (Ref)	-
TT+CT	63.7%	62.2%	65.3%	1.132 (0.748-1.712)	0.558
Recessive					
CT+CC	82.2%	82.1%	82.3%	1 (Ref)	-
TT	17.8%	17.9%	17.7%	0.990 (0.588-1.666)	0.969
Over-dominant					
CC+TT	54.1%	55.7%	52.4%	1 (Ref)	-
CT	45.9%	44.3%	47.6%	1.129 (0.757-1.683)	0.552

Clinical characteristics of participants were stratified by RXRA rs3132294, RXRG rs1128977, and RXRG rs2651860 SNPs. Based on the evidence, no significant correlation between cases and controls was observed in RXRA rs3132294 SNP and RXRG rs2651860 SNP (Tables 6-7).

**Table 6:** Clinical characteristics of participants stratified by RXRA rs3132294 variant ^a^chi-square test, ^b^ANOVA, ^c^Kruskal-Wallis test, ^*^ statistically significant

RXRA rs3132294	Case				Control			
Characteristics	GG	GA	AA	P-value	GG	GA	AA	P-value
Gender (Male)	52.1%	61%	51.5%	0.513^a^	41.3%	45.8%	50%	0.769^a^
Age (Years)	46±11.25	44.43±11.58	52.78±13.29	0.139^b^	34.25±9.61	37.77±9.60	43.85±11.92	0.131^c^
Body mass index (kg/m^2^)	28.17±3.89	27.27±4.19	27.41±5.02	0.436^b^	23.54±4.43	23.65±4.40	23.19±4.55	0.948^b^
Waist circumference (cm)	98.26±11.31	96.96±10.45	97.11±10.25	0.688^c^	84.48±13.12	86.49±12.03	86.58±9.73	0.637^b^
Systolic blood pressure (mmHg)	116.60±10.37	119.36±11.92	126.67±20.62	0.842^c^	114.21±12.90	116.07±8.30	115.77±15.79	0.257^c^
Diastolic blood pressure (mmHg)	77.95±6.95	78.30±7.09	78.89±3.33	0.408^c^	76.98±8.91	75.80±6.16	73.08±9.47	0.313^c^
Fasting blood sugar (mg/dl)	94.43±9.91	94.16±8.41	94.73±11.76	0.978^b^	90.50±7.23	92.83±7.40	92.92±8.53	0.947^c^
Total cholesterol (mg/dl)	246.28±47.72	256.41±80.09	259.82±27.19	0.122^c^	167.98±21.56	163.33±27.17	170.62±22.09	0.448^b^
Triglyceride (mg/dl)	242.91±129.62	239.31±123.11	234.55±152.93	0.638^c^	80.61±31.02	84.22±32.11	74.69±23.89	0.376^c^
HDL-cholesterol (mg/dl)	37.44±10.08	37.24±14.44	38.73±13.40	0.905^c^	51.77±8.53	52.67±10.15	51.69±7.66	0.941^c^
LDL-cholesterol (mg/dl)	144.85±42.39	158.04±77.36	145.09±41.80	0.905^c^	94.73±18.45	94.57±18.97	96.46±18.38	0.945^b^

**Table 7:** Clinical characteristics of participants stratified by RXRG rs2651860 variant ^a^chi-square test, ^b^ANOVA, ^c^Kruskal-Wallis test,^ *^ statistically significant

RXRG rs2651860	Case				Control			
Characteristics	TT	GT	GG	P-value	TT	GT	GG	P-value
Gender (Male)	59.5%	53.2%	57.1%	0.742^a^	47.2%	43.4%	33.3%	0.810^a^
Age (Years)	46.42±12.58	45.62±11.09	42.67±6.50	0.734^b^	36.71±9.96	37.13±10.66	31.0±6.06	0.242^c^
Body mass index (kg/m^2^)	28.11±4.21	27.37±3.87	28.40±4.67	0.513^b^	23.60±4.66	23.50±4.12	21.56±3.59	0.604^b^
Waist circumference (cm)	96.79±11.52	98.34±9.80	97.43±13.84	0.714^b^	86.73±12.45	84.45±12.44	78.20±9.52	0.241^b^
Systolic blood pressure (mmHg)	119.18±13.61	117.58±10.71	117.50±8.80	0.142^c^	115.72±12.17	114.76±10.30	107.50±15.0	0.073^c^
Diastolic blood pressure (mmHg)	78.44±8.19	77.95±5.48	78.33±4.08	0.728^c^	77.39±8.98	74.92±6.43	70.0±0	0.884^c^
Fasting blood sugar (mg/dl)	95.28±9.45	93.33±9.62	97.0±9.06	0.369^b^	91.50±8.15	92.12±6.34	91.60±9.81	0.672^c^
Total cholesterol (mg/dl)	260.74±77.42	242.72±38.75	238.29±29.18	0.685^c^	167.77±20.69	165.52±26.68	166.20±32.01	0.909^c^
Triglyceride (mg/dl)	241.53±144.94	243.08±112.74	206.29±127.66	0.488^c^	87.34±32.88	76.18±27.45	65.80±33.09	0.310^c^
HDL-cholesterol (mg/dl)	37.44±13.63	37.43±9.92	38.0±15.47	0.897^c^	52.59±9.28	51.32±8.81	56.80±12.40	0.262^c^
LDL-cholesterol (mg/dl)	157.22±72.44	143.17±38.62	141.14±40.32	0.938^c^	96.24±17.64	94.29±19.82	87.80±19.27	0.567^b^

Individuals carrying the T-allele of the rs1128977 SNP showed significantly higher levels of body mass index in controls (P < 0.05), higher diastolic blood pressure in cases (P < 0.05), higher waist circumference (P < 0.05), and lower levels of HDL-C in cases and controls (P < 0.05) (Table 8).

**Table 8:** Clinical characteristics of participants stratified by RXRG rs1128977 variant ^a^chi-square test, ^b^ANOVA, ^c^Kruskal-Wallis test,^ *^ statistically significant

RXRG rs1128977	Case				Control			
Characteristics	CC	CT	TT	P-value	CC	CT	TT	P-value
Gender (Male)	46.4%	57.5%	69%	0.127^a^	44.4%	39.4%	57.1%	0.279^a^
Age (Years)	44.73±11.86	46.71±12.10	45.60±10.08	0.688^b^	36.08±11.01	36.13±10.36	38.87±7.38	0.497^b^
Body mass index (kg/m^2^)	27.87±4.48	27.28±3.83	28.78±3.60	0.257^b^	23.90±4.67	22.57±3.81	25.09±4.82	0.030^c*^
Waist circumference (cm)	94.98±11.68	97.81±10.84	101.79±8.43	0.039^b*^	85.34±13.16	83.24±10.54	91.65±14.24	0.021^b*^
Systolic blood pressure (mmHg)	118.05±10.83	116.12±11.04	124.20±14.70	0.264^c^	118.10±11.11	112.90±12.03	114.77±8.79	0.499^c^
Diastolic blood pressure (mmHg)	77.44±6.24	77.84±6.64	80.0±8.04	0.010^c*^	77.10±7.57	74.68±8.19	76.36±5.81	0.671^c^
Fasting blood sugar (mg/dl)	92.06±8.40	95.78±10.47	94.44±8.20	0.113^b^	91.59±7.49	91.22±7.54	93.50±6.88	0.432^b^
Total cholesterol (mg/dl)	255.28±86.56	250.05±40.61	247.85±45.96	0.669^c^	166.45±20.79	166.42±26.56	166.37±24.37	0.877^c^
Triglyceride (mg/dl)	210.87±110.18	243.82±126.56	285.26±153.48	0.167^c^	81.57±31.56	79.92±31.37	85.21±29.96	0.439^c^
HDL-cholesterol (mg/dl)	38.47±9.46	38.71±13.77	32.59±9.34	0.024^c*^	51.29±9.01	54.05±9.55	49±7.62	0.023^c*^
LDL-cholesterol (mg/dl)	153.74±79.97	149.74±41.10	142.44±46.30	0.789^c^	95.25±17.15	94.16±19.94	95.58±19.84	0.930^b^

The results indicated that there was equal SNPs frequency in males and females for rs3132294, rs2651860, and rs1128977 SNPs.

## Discussion

This study examines the association between rs2651860 SNP, rs1128977 SNP, and rs3132294 SNP in RXR genes and dyslipidemia. Our findings suggest that some clinical characteristics, including HDL-C levels, body mass index, waist circumference, and diastolic blood pressure, were associated with rs1128977 SNP (P < 0.05). Previous studies showed that the rs1128977 SNP positively correlated with the development and severity of various disorders, including diabetic retinopathy, schizophrenia, and androgenetic alopecia [13-16]. According to our results, there was no significant association between rs2651860 SNP and rs3132294 SNP, and dyslipidemia or other clinical characteristics. Based on our knowledge, this is the ﬁrst attempt to empirically survey the importance of rs2651860, rs1128977, and rs3132294 variations in dyslipidemia in the Iranian population. 

Extensive studies have been performed to investigate the possible role of RXR gene polymorphisms in different diseases. Sentinelli et al. revealed that RXR gene SNPs could increase the genetic susceptibility of various disorders. Contrary to our results, their outcomes showed that the rs2651860 SNP could be significantly associated with an increased level of Apo-B and LDL-C in familial combined hyperlipidemia patients [10]. Other similar research studies have indicated that other variants including (RXRG) rs2134095 and (VDR) rs2228570 have significant interaction with LDL-C levels [17]. Several studies have shown a positive correlation between SNPs in the RXRG gene and type 2 diabetes. For example, a case-control study of 264 patients in Taiwan reported a significant association between rs1479355 SNP in the RXRG gene and type 2 diabetes [18]. A similar investigation in this area showed that the single nucleotide polymorphism of RXRG rs3753898 might be related to the genetic susceptibility of type 2 diabetes [19]. In addition, Chen et al. worked on other SNPs in the RXRG gene. Their studies revealed that rs3818569 SNP carriers had a lower risk of type 2 diabetes than the reference group [18]. More comprehensive studies were performed to examine other variants of the RXR gene. Vimaleswaran et al.'s findings demonstrated that variations in the RXR gene, including VDR (22 tag SNPs) and RXRG (23 tag SNPs), did not provide strong evidence for interactions between allelic variations in VDR and RXRG genes on metabolic outcomes [20]. The relationship between RXRA polymorphisms and lipid and lipoprotein levels has been widely considered [7, 21-23]. In a study, Lima et al. reported that polymorphisms in the RXRA gene (rs11381416) influence the triglycerides level in a Southern Brazilian population [7]. Inconsistent with our results, in a recent study, Grzegorzewska et al. demonstrated that RXRA SNPs (rs749759, rs10776909) were associated with the prevalence of myocardial infarction but not with serum lipids [2]. Hence, due to fewer investigations on the impact of rs2651860, rs1128977, and rs3132294 polymorphisms in dyslipidemia, this research was accomplished in an Iranian population to evaluate the association between these SNPs and dyslipidemias.

This research was a preliminary report on the effect of SNPs in RXR genes on dyslipidemia in an Iranian population. This study profits from a large cohort of Iranian subjects and determines the role of RXR polymorphisms. Nevertheless, due to the limited number of polymorphisms studied in this experiment, estimating a more significant number of SNPs could help unravel extra comprehensive information concerning RXR gene SNPs and dyslipidemia. Finally, our study was done in a single-center setting and the results could not be generalized to other ethnicities, so more investigations need to be performed on populations with different genetic backgrounds.

## Conclusions

The present study suggests that variation in the RXR genes may contribute to some clinical characteristics. However, more genetics association investigations are required to study the influence of different polymorphisms in the RXR genes on the incidence of dyslipidemia. This investigation increases our information about the association between SNPs in the RXR genes and dyslipidemia, particularly in the Iranian population, and proposes that more studies are required to evaluate the other polymorphisms in a larger cohort of the Iranian people. In this context, in addition to SNPs in RXR genes, transcriptomic estimation of these genes can be another essential point to unravel the prominent role of RXR genes.
